# Editorial Viewpoint on the Special Issue “Food Process Modeling, Optimisation and Control” from Editorial Board Members’ Collection Series

**DOI:** 10.3390/foods14061039

**Published:** 2025-03-19

**Authors:** Mohsen Gavahian, Weibiao Zhou

**Affiliations:** 1 Department of Food Science, National Pingtung University of Science and Technology, 1, Shuefu Road, Neipu, Pingtung 91201, Taiwan; 2 Department of Food Science and Technology, National University of Singapore, 2 Science Drive 2, Singapore 117542, Singapore; weibiao@nus.edu.sg

The guest editors, Dr. Mohsen Gavahian from the National Pingtung University of Science and Technology of Taiwan and Dr. Weibiao Zhou from the National University of Singapore, are pleased to present the editorial overview of the Special Issue entitled “Editorial Board Members’ Collection Series: Food Process Modeling, optimization, and Control”. This Special Issue addressed recent trends in applying mathematical modeling, optimization, and process control to food processing.

Food process engineering plays a crucial role in food production by employing the latest technologies such as Artificial Intelligence (AI)-based mathematical modeling, optimization, and process control. Mathematical modeling, a key element of Industry 4.0, has been used for an in-depth understanding of food processes and improving process efficiency and has been combined with AI and machine learning to address the nonlinear nature of food processing and the wide range of processing parameters. Besides, optimization has been used to maximize production yield and product quality while reducing waste and energy consumption. Furthermore, process control has been utilized to ensure process safety and product quality while reducing wastage and maximizing efficiency. Recent advancements in food process modeling, optimization, and control have significantly enhanced our understanding of complex food systems, enabling improvement of process efficiency and product quality. However, gaps persist in integrating multidisciplinary approaches and advanced technologies to optimize these processes fully. In this context, the guest editors launched a special issue in the editorial board members’ Collection Series entitled “Food Process Modeling, optimization, and Control” to provide a collection of the latest findings in this critical area of research. This special issue attracted the attention of highly reputed researchers and active research teams worldwide. Submitted manuscripts were subjected to peer review, and the most suitable works were selected and accepted for publication, as highlighted in the following.

Hiranpradith et al. contributed to the special issue by a submission on “Optimisation of Ultrasound-Assisted Extraction of Total Phenolics and Flavonoids Content from *Centella asiatica*” [1]. These researchers optimized ultrasound-assisted extraction parameters using response surface methodology (RSM), identifying ethanol concentration and solvent volume as key factors. At the same time, mathematical models were explored for process prediction. The results highlight the capability of the optimization approach to maximize antioxidant-rich compound extraction and the capability of data modeling for process prediction with high confidence.

Namjoo et al. explored “RSM-Based Optimization Analysis for Cold Plasma and Ultrasound-Assisted Drying of Caraway Seed” in another effort to utilize optimization approaches in the food industry [2]. Cold plasma and ultrasound enhanced the hot-air drying of caraway seeds and reduced drying time and energy consumption. The combined plasma-sonication method improved drying efficiency and product quality, with notable increases in total phenolic, flavonoid, and antioxidant content. Optimized conditions achieved relatively high desirability, highlighting the importance of integrating innovative processing technologies and optimization for superior results.

Besides, Punthi et al. explored the “Optimization of Plasma Activated Water Extraction of Pleurotus ostreatus Polysaccharides on Its Physiochemical and Biological Activity Using Response Surface Methodology” and contributed to the optimization of an emerging food processing technology [3]. Applying the innovative extraction process significantly enhanced the yield, and optimizing the process enhanced biological activities such as phenolic content and antioxidant properties. The findings highlight the potential of integrating an emerging technology (plasma-activated water) and optimizing it for the sustainable development of natural antioxidants for food industry applications.

Among the submitted review works to the special issue was “Advances in Designing Essential Oil Nanoformulations: An Integrative Approach to Mathematical Modeling with Potential Application in Food Preservation” by Soni et al. [4]. This review explores strategies to develop encapsulated essential oils, highlighting the role of AI in optimizing nanoemulsion formulations, stability, and release kinetics for enhanced efficacy in food systems. Advanced modeling-based encapsulation methods and their applications in preserving food commodities are also discussed, emphasizing their potential impact on the food and agricultural industries. Raja et al. also contributed to the special issue with a review article on “Modeling and Simulation of 3D Food Printing Systems—Scope, Advances, and Challenges” [5]. This review highlights the advancements in food 3D printing, focusing on modeling techniques to optimize processes and simulate dynamics. It details methods to predict material behavior, modify internal textures, and ensure post-printing stability, aiding printer design based on material and process parameters, underscoring opportunities for computational and data-driven simulations to address challenges and expand applications in food manufacturing.

The Special Issue “Food Process Modeling, Optimization, and Control” contributed to addressing the research gap in integrated process modeling, optimization, and control by presenting research that combines computational modeling, control strategies, and experimental validation to optimize food processing operations. Future research may focus on developing sustainable processing methods, incorporating real-time monitoring and control systems, and leveraging machine learning algorithms to predict and enhance food production. Also, applying advanced optimization, modeling, and process control to up-scale industrial units can practically contribute to achieving the ultimate goal of sustainable food production. Such efforts can contribute to achieving sustainable development goals [6] as outlined by the United Nations [7].

## Data Availability

Not applicable.
